# Treatment of Liver Metastases in Patients with Neuroendocrine Tumors

**DOI:** 10.1155/2012/790635

**Published:** 2012-03-18

**Authors:** Dan Granberg, Wouter de Herder, Dermot O'Toole, Larry Kvols

**Affiliations:** ^1^Division of Endocrine Oncology, Department of Medical Sciences, Uppsala University, SE-751 85 Uppsala, Sweden; ^2^Erasmus University Medical Center, 3015 CE Rotterdam, The Netherlands; ^3^Department of Gastroenterology and Clinical Medicine, St. James's Hospital and Trinity College Dublin, Dublin, Ireland; ^4^H. Lee Moffitt Cancer Center and Research Institute, Tampa, FL 33612, USA

Neuroendocrine tumours may originate from the lungs, thymus, stomach, gastrointestinal tract and endocrine pancreas. A majority of the tumours are malignant. Metastases occur to regional and distal lymph nodes, liver, bones, lungs, mammary glands, subcutaneous tissue, central nervous system and adrenal glands. Although most neuroendocrine tumours are relatively slowly growing, poorly differentiated neuroendocrine carcinomas are fast growing neoplasms with high proliferative activity. 

A characteristic feature of many neuroendocrine tumours is the ability to produce and secrete various hormones and peptides, leading to endocrine symptoms that can be very disabling and cause substantial morbidity. Small bowel carcinoids, for example, produce serotonin giving rise to the classical carcinoid syndrome with flushing, diarrhea, right-sided heart disease and asthma. A prerequisite for the carcinoid syndrome to occur is usually the presence of liver metastases. Lung carcinoids rarely produce serotonin, but may instead secrete histamine causing an atypical carcinoid syndrome with generalized flushing, diarrhea, periorbital oedema, lacrimation and asthma. They may also produce adrenocorticotropic hormone or corticotropin-releasing factor, resulting in an ectopic Cushing's syndrome. Endocrine pancreatic tumours may as well secrete various hormones, such as gastrin, insulin, glucagon, vasoactive intestinal polypeptide (VIP) or somatostatin, resulting in the corresponding syndrome. 

The treatment of patients with metastatic neuroendocrine tumours is based on primary tumour origin, tumour biology, stage and grade and includes debulking by surgery, liver embolization with particles, chemoembolization, radioembolization, radiofrequency ablation and peptide receptor radionuclide therapy (PRRT) with ^90^Yttrium-DOTATOC or ^177^Lutetium-DOTATATE. Medical treatment consists of biotherapy with alpha-interferon and somatostatin analogues, various chemotherapy regimens, angiogenesis inhibitors, tyrosine kinase inhibitors and mTOR inhibitors. In this special issue in the International Journal of Hepatology, focus is on the various specific treatment possibilities for patients with neuroendocrine tumours metastatic to the liver. There are two papers describing the role of surgery in these patients, and one clinical study reporting the results of liver transplantation. Surgical debulking should always be considered, and liver transplantation may in selected cases be an option. Because of the immunosuppression, it is however of utmost importance that every effort is made to exclude remaining tumour outside the liver before transplantation. 

Three papers deals with hepatic arterial embolization, one of them reviews the role of hepatic arterial embolization for debulking of liver metastases. Another paper is about a clinical study and a review of radioembolization, which is a promising alternative for this patient group with possible long-lasting effect and few serious adverse effects. An important disadvantage (also with particle and chemoembolization) is that most patients with neuroendocrine tumours metastatic to the liver in addition have spread of the tumour to lymph nodes and/or other distant organs such as the bones, necessitating systemic therapy. In patients with normal bone marrow and renal function, PRRT is thus often preferred to radioembolization, which however may be considered if the patient shows progression later after PRRT. A randomized clinical trial comparing the various embolization methods, particle embolization, chemoembolization and radioembolization, would nevertheless be highly desirable.

Three papers review the possible systemic therapies for patients with liver metastases from neuroendocrine tumours. This year, two new drugs have been approved for treatment of patients with metastatic endocrine pancreatic tumours, everolimus and sunitinib. This represents an important progress in the therapeutic arsenal for patients with neuroendocrine tumours. There is however still a need for more new drugs, and especially for patients with midgut carcinoids, in whom the therapeutic options are limited after progression. In addition, there is an urgent need to learn how to use, combine and sequence the various therapeutic alternatives, including chemotherapy, biotherapy, newer drugs and PRRT.

*Dan Granberg* *Dan Granberg* *Wouter de Herder* *Wouter de Herder* *Dermot O'Toole* *Dermot O'Toole* *Larry Kvols* *Larry Kvols*

